# Assessing Genetic Divergence and Adaptive Potential of Aroeira (*Myracrodruon urundeuva* Allemão LC, Anacardiaceae) Across Brazilian Biomes

**DOI:** 10.3390/plants15101505

**Published:** 2026-05-15

**Authors:** Marcelo Augusto Mendes Alcantara, Bruno Cesar Rossini, Marcela Aparecida de Moraes Silvestre, Romain Guyot, Andrea Garavito, Patricia Ferreira Alves, Diego Peres Alonso, Paulo Eduardo Martins Ribolla, Mario Luiz Teixeira de Moraes, Celso Luis Marino

**Affiliations:** 1 Department of Genetics, Microbiology and Immunology, Institute of Biosciences, São Paulo State University (Unesp), Botucatu 18618-689, São Paulo, Brazil; 2 Department of Forestry & Environmental Resources, College of Natural Resources, North Carolina State University, Raleigh, NC 27607, USA; 3 Department of Bioprocesses and Biotechnology, School of Agricultural Sciences, São Paulo State University (Unesp), Botucatu 18610-034, São Paulo, Brazil; 4 Institute for Biotechnology, São Paulo State University (Unesp), Botucatu 18607-440, São Paulo, Brazil; 5 UMR DIADE, Research Institute for Development (IRD), University of Montpellier, CIRAD, F-34394 Montpellier, France; 6 CIRAD, UMR AGAP Institut, F-34398 Montpellier, France; 7 UMR AGAP Institut, CIRAD, INRAE, Institut Agro, University of Montpellier, F-34398 Montpellier, France; 8 Department of Crop Sciences, Food Technology and Socioeconomics, School of Natural Sciences and Engineering, São Paulo State University (Unesp), Ilha Solteira 15385-007, São Paulo, Brazil

**Keywords:** Brazilian biomes, genetic diversity, conservation strategies, biome-specific adaptation, NGS sequencing

## Abstract

Genetic diversity is critical for adaptability and resilience in response to environmental pressures, especially considering that human activities have drastically reduced natural populations. The endemic South American species, *Myarcrodruon urundeuva* (aroeira, Anacardiaceae), is found in the Caatinga dry forest, Brazilian Savanna, Atlantic Forest, and Pantanal Wetland biomes and has suffered from population reduction due to overexploitation of its wood. This study analyzed the genetic diversity of *M. urundeuva* across biomes and transition zones in Brazil using ddRADseq sequencing. Samples from 115 individuals and 12 populations were sequenced, generating 1427 informative SNP markers. The average allelic richness was 1227 for the 12 populations, and the inbreeding coefficient (*F_is_*) ranged from 0.001 to 0.196. Notably, the Caatinga dry forest biome populations displayed a highly differentiated cluster. Clustering analysis indicates that genetic diversity patterns from other populations also show distinct population structures with significant differences in its distribution. These findings highlight the need for further research in northeastern Brazil and emphasize the importance of genetic diversity in conservation planning. Therefore, strategies that integrate conservation and molecular analyses are essential to safeguard *M. urundeuva*’s diversity and adaptability.

## 1. Introduction

With the increasing demand for more land for agriculture and grazing, Brazil’s diverse biomes continue to suffer from a reduction in forest areas. As a result, there is a need to assess the genetic diversity of remaining species in the face of climate change and population reduction. As an example, *Myracrodruon urundeuva* Allemão LC (Anacardiaceae) is an endemic South American tree species found in Paraguay, Bolivia, and Argentina, and widely distributed across Brazil’s various biomes, including the Caatinga dry forest, Brazilian Savanna, Atlantic Forest, and Pantanal Wetland [1,2,3,4,5,6]. Popularly known as aroeira, aroeira-preta or aroeira-do-sertão, the species is recognized for its medicinal properties, including antifungal, antioxidant, and anti-bacterial [7,8,9,10,11,12], as well as its high-quality, resistant and dense wood, making it a preferred material for furniture manufacturing and construction [3,11,12,13].

The wide distribution of the species suggests its adaptability to diverse environmental conditions and its ecological significance, particularly in the region known as the ‘Dry Diagonal,’ a cross-country multi-biome zone that splits the continent’s two major rain forests, Amazonia and the Atlantic Forest [14,15]. This zone consists of a belt of arid and semi-arid ecosystems extending from the Paragufayan Chaco to northeastern Brazil [14,16]. It acts as an ecological barrier, limiting gene flow between populations from different biomes and promoting population isolation events that may lead to distinct genetic structure patterns and local adaptation [17,18].

Research has demonstrated the variability in genetic diversity across populations from different biomes, indicating the profound impact that local environment conditions exert on population dynamics [19]. These factors highlight the importance of research on the preservation of genetic variability, especially for native species from highly exploited biomes such as the Brazilian Savanna and Atlantic Forest, which are often targets of real estate development and agricultural expansion [20,21,22,23]. Therefore, understanding the distribution of the genetic differentiation of a species is essential to define effective conservation and management strategies, especially in light of climate change, which is expected to intensify aridity in the affected regions [15,24].

Among many possibilities, DNA sequencing associated with restriction sites (Restriction Digest Associated DNA Sequencing, RADseq and variations) is an important scientific breakthrough in population genomic sequencing methodologies. It enables the identification of a large number of markers, facilitating comprehensive studies on genetic diversity and population structure [25,26,27]. One advantage of the RADseq method is that it does not require any prior genomic information for the taxa under study. Consequently, the technique is widely used to discover and genotype Single Nucleotide Polymorphisms (SNPs). This technique has been shown to offer significant benefits in the ecology and evolutionary study of non-model organisms [25,26,27,28,29,30,31]. In this context, the present study conducted a genetic analysis of *M. urundeuva* individuals across distinct biomes in which the species occurs in Brazil, including ecotone zones between the Brazilian Savanna and Atlantic Forest. The aim of the study was to identify signs of population structure, diversity, and adaptation to different environmental conditions.

## 2. Results

### 2.1. Genomic Library Construction and SNP Identification

A total of 86,040,542 million reads was produced for the 115 sampled individuals of *M. urundeuva*, ranging from 264 to 2,667,829 million reads per sample. These reads were filtered to include only those with a quality of Q > 30, resulting in 42,937,401 million reads, representing about 49.90% of the total. The threshold of missing data for the analyzed loci was 20%. As a result, 27 samples were excluded from the analysis due to missing data (<30%) and insufficient reads (<250,000 reads). The construction of the panel of potential SNP polymorphic sites resulted in an average of 2195 markers for all 88 individuals with a coverage of approximately 15×. After applying stringent filters for data quality and number of reads for 88 individuals, the final panel comprised 1427 SNP markers.

### 2.2. Allele Richness, Private Alleles, and Heterozygosity

Genetic analyses of *M. urundeuva* populations focusing on estimates of private alleles (Supplementary Data S1) and allelic richness (Supplementary Data S2) revealed interesting insights about the species’ genetic diversity and structure across the biomes. The occurrence of private alleles was exclusive to the Caatinga dry forest in Seridó (SE), with a total of 134 alleles. The average allelic richness was estimated at 1227 for all populations. Despite having a significant number of private alleles, the Caatinga population showed the lowest allelic richness with a mean of 1174 alleles, while the ecotone population exhibited the highest mean allelic richness, with 1250 alleles. The Atlantic Forest, Brazilian Savanna, and Pantanal exhibited mean values of 1248, 1227, and 1235, respectively.

The observed (*Ho*) and expected (*He*) heterozygosity values varied among the analyzed biomes (Supplementary Data S3). The Atlantic Forest presented an (*Ho*) of 0.23 and an (*He*) of 0.27, while the Brazilian Savanna showed slightly lower values, with (*Ho*) at 0.21 and (*He*) at 0.26. The Caatinga exhibited the lowest genetic diversity, with an (*Ho*) of 0.13 and an (*He*) of 0.15. In contrast, the ecotone showed the highest values, with an (*Ho*) of 0.24 and an (*He*) of 0.29, indicating greater genetic variability. Finally, the Pantanal exhibited intermediate values, with (*Ho*) at 0.22 and (*He*) at 0.25. These results suggest different levels of genetic diversity across biomes, which are potentially influenced by environmental factors and historical demographic events.

### 2.3. Population Structure and Genetic Diversity Across Biomes

**Figure 1:**
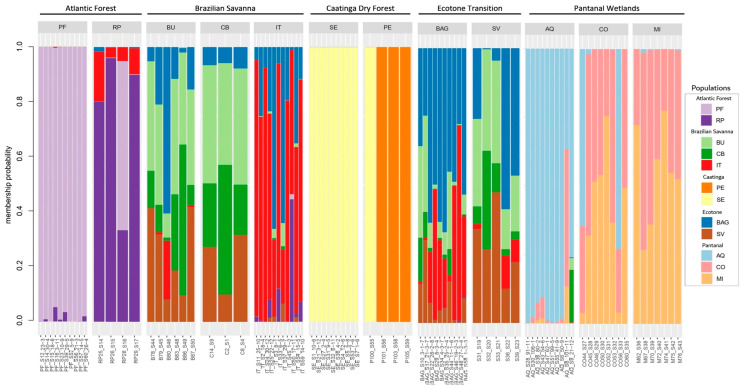
Association of genotypes by population for *M. urundeuva* from the biomes where the species occurs in Brazil. Populations: Atlantic Forest–Paulo de Faria (PF), Ribeirão Preto (RP); Brazilian Savanna–Bauru (BU), Cuiabá (CB), Itarumã, (IT); Brazilian Savana-Atlantic Forest Transition (BAT)–Active Germplasm Bank (BAG), Selvíria (SV); Caatinga dry forest–Petrolina (PE), Seridó (SE); Pantanal Wetlands–Aquidauana (AQ), Corumbá (CO), Miranda (MI).

The genetic diversity of *M. urundeuva* (Figure 1) exhibited distinct structuring clusters among populations associated with the Caatinga dry forest, the Atlantic Forest, and the Pantanal Wetland. For the populations from the Brazilian Savanna and the ecotone transition zones, a mixed cluster was identified, indicating that these populations may have experienced significant reproductive interaction and allele exchange over several decades.

**Figure 2:**
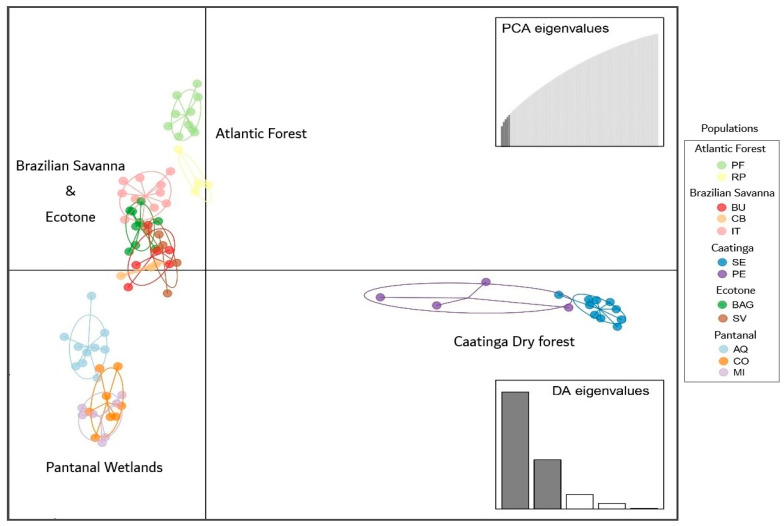
Correlation by discriminant analysis of principal components (DAPC) for *M. urundeuva* from the biomes where the species occurs in Brazil. Populations: Atlantic Forest–Paulo de Faria (PF), Ribeirão Preto (RP); Brazilian Savanna–Bauru (BU), Cuiabá (CB), Itarumã, (IT); Brazilian Savana-Atlantic Forest Transition (BAT)–Active Germplasm Bank (BAG), Selvíria (SV); Caatinga dry forest–Petrolina (PE), Seridó (SE); Pantanal Wetlands–Aquidauana (AQ), Corumbá (CO), Miranda (MI).

Population structure was analyzed using discriminant analysis of principal components (DAPC, Figure 2) and principal component analysis (PCA, Supplementary Data S4). Both approaches revealed distinct genetic groupings corresponding to populations from the Caatinga dry forest (Seridó, SE; Petrolina, PE), Atlantic Forest (Paulo de Faria, PF; Ribeirão Preto, RP), and Pantanal Wetland (Aquidauana, AQ; Corumbá, CO; Miranda, MI). In contrast, populations from the Brazilian Savanna (Bauru, BU; Cuiabá, CB; Itarumã, IT) and the ecotone (Rosana, BAG; Selvíria, SV) showed similar inter-population relationships across DAPC, PCA, and structure analyses, indicating a more continuous genetic gradient within these biomes.

**Figure 3:**
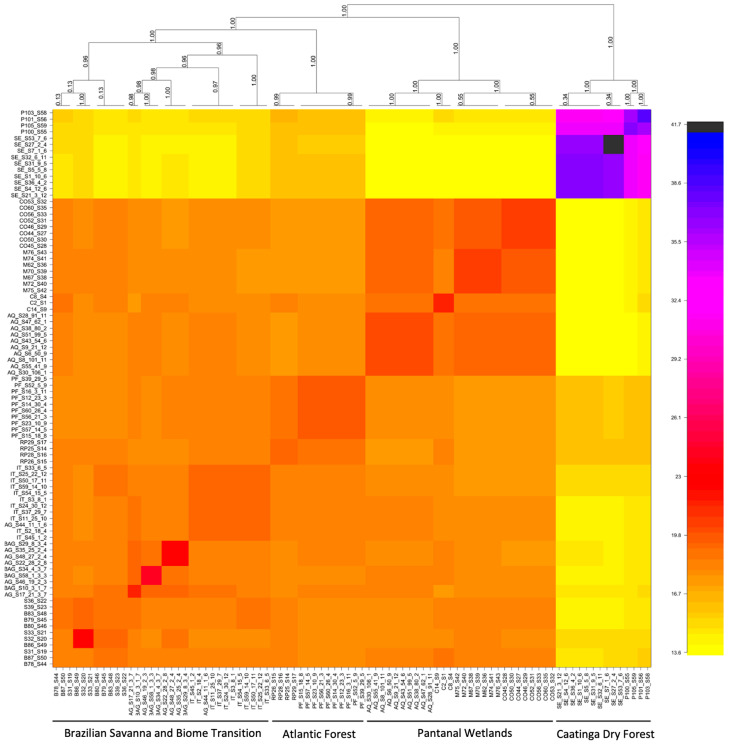
Population structure revealed by FineRADstructure software with the identification of at least four genetic clusters. Colors indicate the correlations between samples, ranging from blue (more correlated) to yellow (less correlated) for *M. urundeuva* from the studied biomes in Brazil.

The haplotype coancestry analysis conducted using the fineRADstructure software (Figure 3) further supported this pattern, revealing at least four main genetic clusters: Caatinga dry forest populations (SE and PE); Pantanal Wetlands (AQ, MI, CO); Atlantic Forest (PF and RP); and Brazilian Savanna/ecotone (BAG, BU, CB, IT, and SV).

This pattern is corroborated by the Mantel Test (Supplementary Data S5), which detected a pronounced genetic distance between the SE and PE populations relative to the others. This indicates strong genetic distinctiveness within the Caatinga dry forest biome. The Analysis of Molecular Variance (AMOVA, Supplementary Data S6) further quantifies this structure, with 28.79% of the total genetic variation distributed among populations, which is consistent with differentiation across the analyzed biomes.

**Figure 4:**
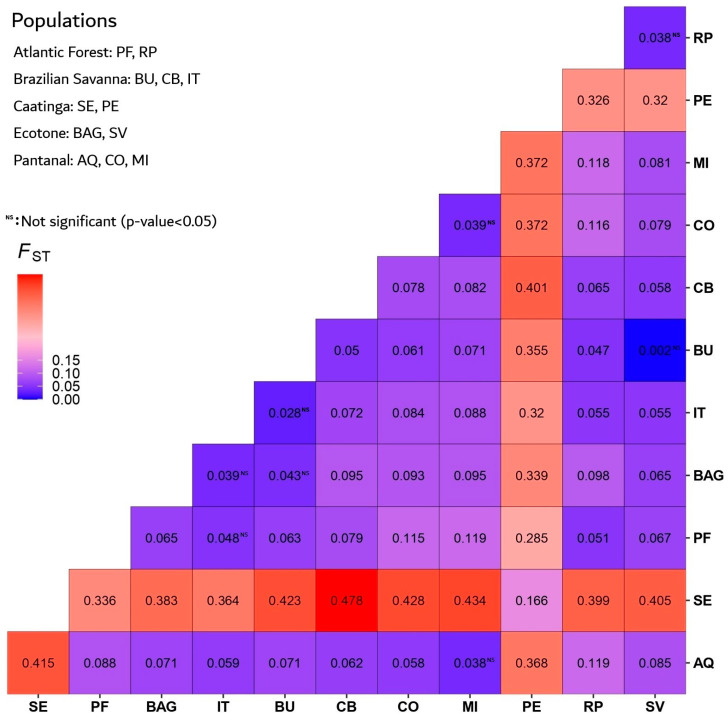
Fixation Index (FST) for *M. urundeuva* from the biomes where the species occurs in Brazil. Populations: Atlantic Forest–Paulo de Faria (PF), Ribeirão Preto (RP); Brazilian Savanna–Bauru (BU), Cuiabá (CB), Itarumã, (IT); Brazilian Savana-Atlantic Forest Transition (BAT)–Active Germplasm Bank (BAG), Selvíria (SV); Caatinga dry forest–Petrolina (PE), Seridó (SE); Pantanal Wetlands–Aquidauana (AQ), Corumbá (CO), Miranda (MI).

Wright’s Fixation Coefficient (FST) [32] (Figure 4) showed that most populations are significantly different from each other. Populations from the Brazilian Savanna (BU, CB, IT) and the ecotone (BAG, SV) displayed low genetic differentiation, with several non-significant pairwise comparisons (*p* > 0.05), such as BU-SV (FST = 0.002) and IT-PF (FST = 0.028). Pantanal populations (AQ, CO, MI) similarly showed moderate to low differentiation, with non-significant comparisons including MI-CO (FST = 0.039) and MI-AQ (FST = 0.038). In contrast, Caatinga populations (SE, PE) exhibited the highest differentiation across all comparisons, with elevated FST values such as SE-MI (FST = 0.434), SE-CO (FST = 0.428), SE-CB (FST = 0.478), and PE-CB (FST = 0.425). These results indicate strong genetic separation of Caatinga populations relative to the other analyzed biomes.

### 2.4. Genetic Differentiation and Environmental Influence on Population Structure

The correlation analysis between populations and climatic variables using PCoA (Supplementary Data S7) showed associations between genetic structure and the biomes of origin. Caatinga dry forest populations were separated from the others and associated with Mean Annual Temperature, Mean Temperature of the Driest Quarter, Mean Temperature of the Warmest Quarter, Isothermality, and Precipitation Seasonality.

Populations from the Brazilian Savanna and ecotone showed positive correlations with Mean Diurnal Range, Temperature Seasonality, Annual Precipitation, Precipitation of the Driest Month, Precipitation of the Driest Quarter, and Precipitation of the Coldest Quarter. Pantanal Wetland populations (AQ, CO, MI) clustered near variables related to Rainfall and Temperature Seasonality. Atlantic Forest populations (PF, RP) were associated with Elevation, Precipitation of the Wettest Month, and Precipitation of the Wettest Quarter.

Outlier detection using pcadapt and Redundancy Analysis (RDA) identified loci associated with environmental variables. The pcadapt analysis detected 106 candidate loci (q < 0.1), and RDA identified only 3 loci (*p* < 0.001), with none SNPs shared between the two approaches. These SNPs were associated with temperature-related variables, Isothermality, Mean Annual Temperature, Temperature of the Warmest Quarter, and Annual Temperature Range, and precipitation-related variables, including Precipitation Seasonality, Precipitation of the Driest Month, Annual Precipitation, and Precipitation of the Wettest Month. Associations with Elevation were also observed.

## 3. Discussion

### 3.1. High-Resolution Population Genetics of M. Urundeuva: Insights into Genetic Structure and Diversity

The genetic distribution of populations, whether in structured or panmictic populations, and the analysis of this distribution across biomes, is highly significant for the genetic conservation of all species. This study is the first to estimate the genetic diversity of *M. urundeuva* using SNP markers on a large scale and across multiple biomes. As such, the results indicate at least four clusters of population structuring based on over 1000 molecular markers that is consistent with the species distribution across Brazil. Moreover, the identification of a large number of markers has enabled a catalog of robust SNPs, demonstrating potential for monitoring species in terms of population genetics and distribution patterns. In this sense, although populations are susceptible to founder effects and cultivation conditions, ex situ conservation of populations originating from the biomes in which the species occurs represents a significant source of genetic variability from populations that are disappearing from their natural regions, thus offering an alternative for species maintenance.

Studies on Brazilian species, including those threatened with extinction such as *Araucaria angustifolia*, highlight the usefulness of SNP markers in assessing population genetic structure across broad geographic ranges, as demonstrated by initiatives to develop SNP chips [19]. Similar efforts have resulted in SNP arrays for species of economic interest including cassava, coffee, and eucalyptus. Complementarily, reduced-representation sequencing approaches such as RAD-Seq have also been applied to several native tree species, including *Dipteryx* (cumaru) [33], *Hymenaea* (jatobá) [34], and *Handroanthus* (ipê) [35], providing valuable datasets for population genetics and conservation. The recent assembly of high-quality reference genomes, such as the purple ipê (*Handroanthus impetiginosus*) [36], enables substantial expansion of SNP discovery. Reference genomes facilitate the alignment of new sequencing reads and the systematic identification of variants, offering a scalable platform that can be extended to additional native species. In this context, expanding genomic resources across Brazilian biomes is likely to accelerate comprehensive assessments of genetic diversity and adaptive structure in currently underrepresented taxa. Moreover, the dataset generated in this study may support future development of SNP panels specifically designed for *M. urundeuva* and other Anacardiaceae species. Such tools could have practical applications in environmental monitoring, forensic timber identification, and conservation programs aimed at mitigating illegal harvesting and preserving genetic diversity across its distribution range.

Due to the lack of studies on similar native trees, the number of loci identified can be considered satisfactory with a mean coverage of 15x. When compared to ddRADseq studies for other tree species with published complete genomes, such as *Populus* and *Eucalyptus* with genome sizes between 0.43 and 0.69 Gb, predictive numbers of SNP markers range from 6.57 to 14.6 thousand loci, respectively [37]. Although higher than the present study, these values are representative of known and cataloged complete genomes, with ample data availability. Thus, even though they represent approximately 1.6% of the total number of loci identified, the 1427 SNPs identified after filtering demonstrate success in the use of the technique. It is also important to consider that the analysis was based on a de novo strategy, i.e., without a reference genome. This involves the identification of common regions among at least 70% of the analyzed individuals and the challenge of DNA extraction from samples that are free of contaminants, a characteristic of the species as it has a high content of secondary compounds.

In aroeira, early studies using microsatellites and other marker systems, such as AFLP and RAPD, have reported genetic differences among populations from distinct regions and biomes [38,39,40,41,42,43,44]. The results obtained here using SNP markers show a convergence between population genetic structure and environmental/climatic patterns across biomes. Studies at this resolution remain essential, especially because *M. urundeuva* does not yet have a published nuclear reference genome or annotated gene regions of functional interest. A first step toward filling this gap was achieved by publishing the complete chloroplast genome of the species, marking the beginning of a new phase in molecular and genomic investigations on *M. urundeuva* [45].

Population structure in forest species encompasses characteristics such as age, size, geographical distribution, and genetic diversity. These factors and characteristics can offer a detailed view of their dynamics and health [46,47,48,49,50]. Also, understanding what these data provide is essential information for the conservation and management of species in their natural habitats [46]. Population structure plays a central role in population genetics studies, as it directly influences genetic diversity and allele distribution. These factors have an impact on the ability of species to adapt and survive environmental changes and various stresses [51,52], where genetically diverse populations have a greater capacity to adapt to environmental changes. The presence of a wide variety of alleles means that there is a greater probability of some of these alleles conferring adaptive advantages in variable environments. Furthermore, allelic richness is an indicator of the health and vitality of a population [53]. Populations with low allelic diversity may be subject to inbreeding problems, genetic drift, and strong homozygosity, and have a poorer ability to adapt to new environmental conditions or threats, such as disease or climate change [54,55]. In a study on *Casearia sylvestris* (guaçatonga) with natural and reforested populations in the Atlantic Forest, the allelic richness for natural populations was on average 1.41 [56]. Although the average allelic richness values found for aroeira populations in the different biomes studied herein are lower (1.161–1.269), they indicate evolutionary potential for the species.

In temperate climate species like *Quercus*, SNP marker evaluation identified a maximum of 30 private alleles per population [57]. For Brazilian species such as palms, values of up to 40 alleles have been found per population when assessing around 1000 markers [58].and these values did not exceed 28 private alleles (10 markers and over 500 individuals) with the use of microsatellite markers [59]. Meanwhile, in tree species like *Dalbergia nigra* (Brazilian rosewood) using microsatellite markers (10 markers, 140 individuals), the average private alleles among populations do not exceed 1.44 [60]. Thus, the values found in this study are above average in one population (Seridó), but present genetic homogenization in the other populations. This suggests significant genetic differentiation in the Caatinga dry forest compared to other biomes for *M. urundeuva*. Such divergence of this population suggests unique factors like evolutionary events, geographical barriers, or limited gene flow in the Caatinga dry forest, possibly influenced by specific environmental factors of this semi-arid biome.

### 3.2. Caatinga as a Genetically Divergent Biome

Reduced levels of heterozygosity may indicate disturbances in forest fragments when evaluating natural populations or a deficit of sampled and/or collected individuals when evaluating progeny and provenance tests. The assessment of genetic diversity is widely used to evaluate the maintenance of natural populations [61,62]. The genetic differentiation among *M. urundeuva* populations revealed by DAPC, fineRAD, as well as AMOVA analysis, highlight the complex evolution and plasticity of the species in different biomes. In general, the analyses indicate that there are significant differences between the Caatinga and the other analyzed biomes. Furthermore, the population structure indicates homogeneity for transition zones and the Brazilian Savanna and less differentiation for the Atlantic Forest. This interaction could be facilitated by several factors, including geographical proximity, ecological connectivity, or historical gene flow events. Further studies on this plasticity are needed to understand the population dynamics within the species, its gene flow, and ecotones [38].

The similarity between the values of observed heterozygosity (*H_o_*) and expected heterozygosity (*H_e_*) highlight the stability of the populations in terms of Hardy–Weinberg Equilibrium (HWE). These values may indicate variability within the natural populations of this study, random mating, and a potentially adequate population size [63,64]. In the literature, authors also report heterozygosity values close to HWE for *M. urundeuva* from natural populations and in progeny tests from various biomes where the species occurs [38]. The authors obtained average values around 0.7 for both (*H_o_*) and (*H_e_*) in the Brazilian Savanna, Atlantic Forest, and Pantanal Wetland. However, for the Seridó population, a study using microsatellite markers found values close to 0.537 for observed heterozygosity, in contrast with 0.567 for expected heterozygosity [38].

From a metabolic and morphological perspective, the impact that the Caatinga climate has on local species, including *M. urundeuva,* may indicate that the species has undergone adaptations when compared to populations found in other biomes [65,66]. Characterized as a semi-arid climate by Köppen since 1918 [67], this region experiences long periods of drought, high temperatures, and irregular rainfall distribution, presenting considerable challenges for the survival of plant species [19,68,69]. The climate in Petrolina, Seridó, and other Caatinga regions is marked by a prolonged dry season, lasting approximately eight months, followed by a relatively short but intense rainy season. This seasonal asymmetry places increased stress on vegetation, especially those adapted to thrive in arid conditions. The predominant northeast winds further exacerbate the aridity, contributing to a distinctive microclimate. The genetic differentiation of the Caatinga dry forest population highlights the need for conservation strategies adapted to this specific group [24]. Strategies that consider the genetic and environmental particularities of the Caatinga dry forest may be crucial for preserving the diversity of *M. urundeuva* in this semi-arid biome, especially the species’ adaptation to the region’s saline soil and inconsistent rainfall patterns during the rainy season [70].

Similarly, Pantanal Wetland populations formed a distinct cluster from the others, suggesting the need for tailored species conservation actions for these populations and the biome more generally, in the states of Mato Grosso do Sul and Mato Grosso, Brazil, and the Paraguayan Chaco where the species is also present. On the other hand, the genetic proximity among Brazilian Savanna populations, as well as the transitions from Brazilian Savanna to Atlantic Forest, suggest a close adaptive history and intense genetic exchange between these regions, mainly between the Brazilian Savanna and Atlantic Forest in the state of São Paulo, a location with a strong transition between both [71]. These factors may indicate common or similar measures for species conservation in these habitats, as the genetic and adaptive plasticity of the populations, although diverse, still exhibit similarities.

Although similar, there are differences among the Atlantic Forest populations and compared to the other biomes. These populations showed convergence with Brazilian Savanna populations and the ecotone, forming a cluster in PCA. However, greater differences were highlighted by DAPC and fineRAD analysis, indicating that the Atlantic Forest biome is a genetic cluster different from other biomes. This proximity suggests gene flow among these populations over time, and it is plausible that factors such as habitat connectivity or dispersal events have contributed to this genetic similarity [72,73]. Anthropogenic factors may also be contributing to the proximity of the Atlantic Forest and Brazilian Savanna biomes, especially in their ecotones, as these biomes are highly degraded, with about 50% of the original area remaining for the Brazilian Savanna [74,75] and less than 10% for the Atlantic Forest [61]. This reduction in individuals in each habitat may lead to directional mating and consequent genetic homogenization at the population level. This was also demonstrated by the *F_st_* test, with several values approaching the threshold of statistical non-significance. Further studies on gene flow at the biome level and transition zones could shed light on this issue. Although the exploitation of aroeira is recent in its evolutionary history, it is worth noting that anthropogenic interference is responsible for significant damage to various species at the population level, whether through harvesting, predation, deforestation, or regional or global climate change [52,76].

The Brazilian Savanna populations occur in the geographic center of the county and are surrounded by the other national-scale biomes. As such, these populations may be acting as a zone of homogenization. Other studies on *M. urundeuva* populations from Argentina, Paraguay [77], and Brazil [38] also indicated significant population diversity and differentiation between the biomes in which the species occurs. Finally, populations with low *F_st_*, although statistically significantly different, may not exhibit biologically meaningful differentiation [64]. This suggests genetic proximity among the Pantanal Wetland populations.

Finally, the genetic variations found between the Northeast and Central-Southeast regions of Brazil corroborate previous results based on SSR and chloroplast markers. Furthermore, this variation reached *F_st_* values up to 0.478, an enormous variation indicating strong population structuring or even signals of possible cryptic species. However, there is a lack of significant evidence from morphological analysis of the populations, in addition to the need to include populations from the states of Minas Gerais and Bahia, factors that could better indicate this distribution.

### 3.3. Climatic Drivers and Environmental Pressures on M. urundeuva Genetic Differentiation Within Biomes

From the perspective of population adaptability across the studied biomes, particularly in the Caatinga, genetic differentiation can be seen through selected markers for resistance to drought, low or no rainfall, and high temperatures, as indicated by multivariate analyses. Temperature variations in the Caatinga dry forest populations (e.g., Seridó) are notable, with daytime temperatures often exceeding 30 °C, and significantly cooler nights. This diurnal temperature range has implications for plant physiology and adaptation strategies. Additionally, the irregularity of rainfall patterns requires that drought-resistant species are assessed for use in conservation initiatives. Their adaptive strategies may indicate them as priority species that are well-suited to the region’s climatic challenges. A previous study simulating natural growth of *M. urundeuva* in semi-arid regions demonstrated this adaptation through the association of physiological growth parameters and response characteristics to water stress [78]. By assessing severe water regimes and observing seedling development, the authors corroborate the findings presented herein for the Petrolina and Seridó populations in the Caatinga biome. SNPs were associated with the climatic variable average temperature in the wettest quarter, while genes were related to U-box domain-containing protein 4-like, which participate in the degradation of regulatory proteins, such as those involved in the cell cycle, cell signaling in response to stress and extracellular signals, morphogenesis, secretory pathway, DNA repair, and organelle biogenesis [79]. Furthermore, the autophagy-related protein 13b gene is related to the recycling of cellular materials, such as cytoplasmic material, nucleic acids, protein complexes, lipid bodies, and damaged organelles [80]. Such genes should be further investigated, since there are regions associated with other variables related to chloroplasts and mitochondria.

Understanding population dynamics is necessary, as there is a lack of data for a large number of native forest species. This poses challenges for conservation in the face of climate change, large-scale environmental impacts, and human alteration of natural areas. Further studies on the association between native populations and their respective biomes are also required, enabling a better understanding of species and environment interactions. Such analyses can provide greater insight into the adaptation of these species to the local climate and offer possible inferences of population dynamics, center of origin, and evolution.

Additionally, one of the key evolutionary modulators for species in these biomes, known as the “Dry Diagonal” of Brazil, is an extensive ecological transition zone stretching from the Northeast to the Midwest, encompassing the Caatinga dry forest, Brazilian Savanna, parts of the Pantanal Wetland, and entering into the Paraguayan Chaco [14,16]. This region is characterized by low annual rainfall and high hydric seasonality, with semi-arid regions receiving less than 800 mm of precipitation per year [18,81]. In these environments, water scarcity imposes significant challenges for plant species, necessitating morpho physiological adaptations for survival. It is important to consider that native forest populations within the Dry Diagonal, face considerable challenges due to adverse climatic conditions, particularly the combined effects of temperature, water deficits, and irregular rainfall patterns, which directly influence population dynamics [16,17]. Natural wildfires during the dry season also play a crucial role, affecting key processes such as seed germination, growth, and reproduction, as observed in the Brazilian Savanna [15,82]. Additionally, habitat fragmentation caused by anthropogenic activities exacerbates genetic isolation among populations, compromising genetic diversity and species resilience to environmental changes. Some authors suggest that categorizing the Dry Diagonal requires analyzing its subregions, as each has distinct ecological characteristics. The Caatinga dry forest, predominantly located in the Northeast of Brazil, consists of xerophytic vegetation adapted to prolonged droughts, including deciduous species and cacti [14,15]. The Cerrado, in the Brazilian Central Plateau, features savanna formations ranging from open grasslands to dense woodlands and harbors exceptional biodiversity. The Pantanal, despite being renowned for its wetlands, also comprises dry-adapted vegetation, particularly along its margins [83].

As previously discussed, it is important to highlight that studies by various authors describe potential adaptations to these extreme conditions [84]. Young individuals of *M. urundeuva* subjected to water deficit exhibited reduced photosynthetic rates and stomatal conductance, indicating a water-use conservation strategy. However, they maintain the integrity of the photosynthetic apparatus, suggesting efficient protective mechanisms against photo-oxidative damage [85]. The accumulation of proline in leaves contributes to osmoregulation, helping sustain cellular water potential. Furthermore, the species demonstrates a high capacity for post-stress recovery, rapidly restoring relative water content and photosynthetic activity upon rehydration. This functional resilience is a critical adaptive trait for coping with the hydric variability of the Dry Diagonal [86]. During early development, seed germination and seedling vigor are highly dependent on soil moisture. Water deficits can hinder root growth and leaf formation; however, morphological and anatomical adaptations, such as reduced leaf area and increased stomatal density, help minimize water loss through transpiration [78,87].

These physiological responses, together with the findings of this study, suggest that *M. urundeuva* possesses a suite of adaptive traits conferring tolerance to the hydric stress typical of the Dry Diagonal, particularly the pressures in the Caatinga dry forests. Understanding these mechanisms is essential to inform conservation strategies and the sustainable management of this species, especially in restoration programs for degraded semi-arid regions in Brazil. In this sense, the application of “adaptive association” is extensive and necessary, as it can inform the conservation of threatened species or can be used for commercial purposes, genetic improvement, cultivar breeding, or reforestation in ex situ areas for species of interest. Adaptive association is based on the understanding and identification of functional loci and the quantification of genetic contributions to complex adaptive traits. Thus, understanding the fundamentals and applications of adaptive association is essential to interpret genetic data in a context of environmental and evolutionary changes [88,89,90].

As long as populations can maintain sufficiently high levels of fertility and genetic variation, the ability to adapt to rapidly changing climates may be sufficient to maintain trees across the landscape, although these populations may experience some delay in adaptation over several generations [91]. Generalized species with high fecundity occurring in large populations are likely to be able to adapt to climate change in relatively few generations and survive during this period, as major competitors will face the same fate of short-term poor adaptation. High rates and distances of seed and pollen dispersal will also positively contribute to their adaptability and migration [91]. On the other hand, species occurring in small and fragmented populations, or those with low fecundity or late sexual maturity, reproductive characteristics more typical of later successional species and high-altitude habitats, are likely to experience greater adaptive delay [91]. Due to its wide geographical distribution, *M. urundeuva* demonstrates climatic and environmental plasticity to withstand forthcoming adaptive challenges. Studies on reforestation and acclimatization of the species in different biomes are needed to verify this theory. Nevertheless, the literature enables us to infer about the adaptability of the species under adverse growth conditions typical of their biomes of origin [92,93,94,95,96,97,98,99,100,101].

### 3.4. Conservation in Light of Genetic Divergence and Implications for Non-Model Species

Research on population genomics and speciation [102] is fundamental, as they can provide more conclusive understanding of the mechanisms acting in the speciation and radiation of forest populations. Regarding *M. urundeuva,* when combined with future research on regions associated with adaptability, genes related to disease or stress resistance, and the complete genomic mapping of the species, the findings of this study could contribute to a better understanding of diversity patterns in the respective biomes of the species occurrence, their adaptations, and possible future speciation resulting from climatic influences. Although, SNP polymorphism data allow us to infer about population diversity and differentiation, the ability to detect levels of selection at play is limited due to various factors, one of which is low polymorphism density within a single species [102]. The authors also highlight that these issues can be improved by analyzing polymorphism across multiple related species and combining polymorphism and divergence data into a single analysis.

An analysis of this scope is possible, as *M. urundeuva* shares its genus with *Myracrodruon balansae* within the Anacardiaceae family. This species occurs in the Pampas in southern Brazil, Argentina, and Paraguay [103], and has close kinship with *Astronium fraxinifolium*, popularly known as gonçalo-alves, and *Astronium graveolens*, known as guaritá. All these species were previously classified under the genus *Astronium* and later subdivided into subgenera *Myracrodruon* and *Astronium* [104]. Thus, there is high probability of genetic proximity between the genera and the potential for sharing of common markers across the species.

## 4. Materials and Methods

### 4.1. Materials

**Figure 5:**
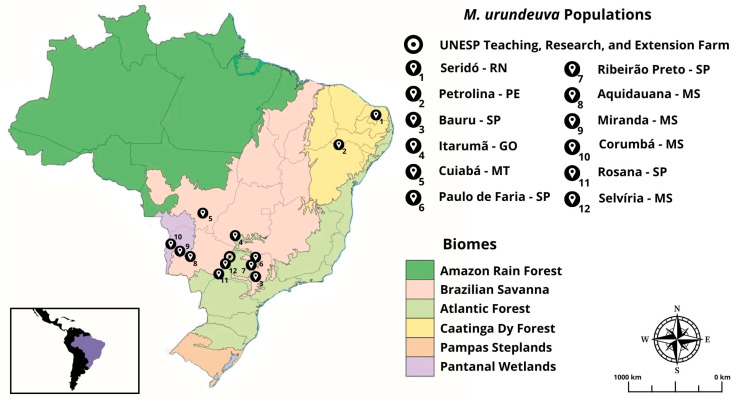
Location of original natural populations in their respective biomes from collected matrices for the installation of *M. urundeuva* progeny tests installed ex situ at the Teaching, Research, and Extension Farm (FEPE), Ilha Solteira Faculty of Engineering (FEIS), Selvíria, Mato Grosso do Sul, and at the Active Germplasm Bank (BAG), an in situ conservation area, in the municipality of Rosana, São Paulo. State Acronyms: RN—Rio Grande do Norte; PE—Pernambuco; SP—São Paulo; GO—Goiás; MT—Mato Grosso; MS—Mato Grosso do Sul.

Leaf samples were collected from *M. urundeuva* individuals cultivated in conserved ex situ progeny and provenance tests established from four biomes: Caatinga, Cerrado, Atlantic Forest, and Pantanal. Thus, these samples represent natural populations originating from the different biomes where the species occurs. The progeny tests are installed at the Teaching, Research, and Extension Farm (FEPE) of the Ilha Solteira School of Engineering (FEIS), São Paulo State University (UNESP), located in the municipality of Selvíria, Mato Grosso do Sul, Brazil (Table 1). Additionally, leaf samples were collected from individuals in the ecotone between the Brazilian Savanna and the Atlantic Forest, located on the border between the states of Mato Grosso do Sul and São Paulo. This area is an in situ conservation site established as an Active Germplasm Bank (BAG), in the municipality of Rosana, São Paulo. A total of 115 individuals were collected for this study, from 12 populations (Figure 5). Each biome is described in Table 2.

### 4.2. Methods

#### 4.2.1. DNA Extraction and Library Preparation for Double Digest Restriction Associated DNA Sequencing (ddRADseq)

Genomic DNA extraction followed an adapted protocol using 3% Cetyltrimethylammonium Bromide (CTAB) method [111]. Quantification was subsequently performed using a NanoDrop ND-1000 Spectrophotometer (NanoDrop Products, Wilmington, DE, USA), and DNA integrity was verified on 1% agarose gels with TBE buffer (Tris/Borate/EDTA) (1×) with a constant voltage of 120 V for one hour.

For the preparation of sequencing libraries, a protocol for ddRADseq [27,112] was followed starting with 500 ng of DNA. Digestion was performed using EcoRI and MspI restriction enzymes (New England Biolabs, Ipswich, MA, USA). and selection of fragments between 350 and 500 bp. Samples were indexed using Nextera XT v2 kit (sets A and B). All samples were purified using magnetic beads ProNex^®^ Size-Selective Purification System kit (Promega Corporation, Madison, WI, USA), quantified using Qubit™ dsDNA HS Assay Kit (Thermo Fisher Scientific, Waltham, MA, USA), and the final pool size checked by High Sensitivity D1000 ScreenTape Assay for TapeStation Systems (Agilent Technologies, Santa Clara, CA, USA) Details of the procedures and adaptations can be found in previous publications [112]. The libraries were sequenced with single-end 150 bp reads using the Illumina Nextseq500 platform (Illumina, San Diego, CA, USA).

#### 4.2.2. Data Analysis: Read Processing, SNP Identification, Genetic Diversity, and Climate Association

To assess the quality of raw data, the FastQC v0.12.1 program [113] followed by STACKS v2.71 software [114] were used to identify SNP loci in sequenced individuals. The denovo_map.pl module was used as there is no reference genome available for *M. urundeuva*. All individuals were treated as a single population to minimize missing data. The filtered and analyzed data were exported with parameters set to r70 and -min-maf 0.05. To build the reference catalog in the denovo_map.pl module, several tests were conducted [115] with *m* ranging from 3 to 8, *M* ranging from 1 to 8, and *n* ranging from 1 to 8 [114,116]. Linkage disequilibrium was evaluated with PLINK v1.07 [117].

For statistical analysis, specific R software v4.3.1 (R Core Team), environment packages and well-established methods were employed. Discriminant Analysis of Principal Components (DAPC) was performed using the “adegenet” package v2.0.0 in the R environment [118]. Additionally, Principal Component Analysis (PCA) was conducted to ordinate the data, which is a widely used method across various fields, including ecology, with statistical tools available in R [119]. Sample by pop assignment were plotted using ggplot2 v3.4.3 [120]

The fineRADstructure software [121] was used to identify population structure based on nearest neighbor haplotype coancestry, calculating the closest haplotypes and assembling a coancestry matrix, using default parameters (−x 100,000 −y 1,000,000 −z 1000). Furthermore, Analysis of Molecular Variance (AMOVA) was conducted using the “poppr” package v2.9.4 [122] to assess genetic differentiation among *M. urundeuva* populations.

The Mantel Test was used to assess the relationship between genetic and geographical distances, contributing to a comprehensive understanding of the species population structure. This isolation-by-distance analysis was based on geographical distances in kilometers and genetic distance from the fixation index among populations, using the “vegan” package v2.6-4 [123] for Mantel and “StAMPP” package v1.6.3 [124] for *F_st_* estimates between populations.

The fixation coefficient (*F_st_*) [125] was calculated to evaluate genetic differentiation among subpopulations, while allelic richness and private alleles were used to quantify the genetic diversity, highlighting unique alleles in each subpopulation. Additionally, observed and expected heterozygosities were calculated to assess observed and expected genetic variability in *M. urundeuva* populations. These datasets were obtained using the “hierfstat” package v0.5-11 [126] in R, with 1000 bootstraps.

To identify loci potentially involved in adaptation, two methods were employed: principal component analysis with the “pcadapt” package v4.3.3 [127]; and Redundancy Analysis (RDA) using the “psych” v2.2.9 [128] and “vegan” v2.6-4 [123] packages in R. This multivariate statistical technique allowed us to explore the relationships between environmental and genetic factors influencing the *M. urundeuva* population structure.

Nineteen climatic variables were extracted from the WorldClim database [129] and plotted with geographical coordinates in QGIS software v3.32.3. Correlated variables (values greater than 0.80) were removed from the analysis. For each analyzed individual, data were exported based on its geographical coordinates from the original population and then analyzed in terms of genetic diversity and correlation with climatic variables. This was done to select SNPs that were highly related to one or more variable as potential loci of local adaptability of the analyzed population. Principal Coordinates Analysis (PCoA) was conducted using data provided by WorldClim [129] allowing spatial visualization of climatic variations and their influence on the distribution of the studied populations. All climatic variables used for the analysis are included in Supplementary Data S7.

## 5. Conclusions

The analyzed *M. urundeuva* populations of the Caatinga, Brazilian Savanna, ecotone, Atlantic Forest, and Pantanal biomes exhibited substantial genetic variability, indicating the influence of region-specific environmental and historical drivers. Caatinga populations showed the highest levels of genetic differentiation, consistently forming a distinct cluster relative to the other biomes. In contrast, populations from the Brazilian Savanna and Atlantic Forest presented greater genetic similarity, suggesting historical connectivity or ongoing gene flow between these regions.

Levels of observed heterozygosity were consistent across different analyses for the species and reflect the capacity of *M. urundeuva* to cope with environmental pressures. The correlations found between genetic patterns and climatic variables also indicate that populations have the potential to respond to local environmental conditions, offering perspectives on the role of climate in shaping adaptive genetic variation and response to climate change.

The pronounced divergence observed for the Caatinga populations highlights the need for broader sampling in the northeastern region of Brazil to better characterize the genetic patterns in this biome. These findings emphasize the importance of considering both genetic structure and local adaptation for native species conservation and management strategies, with the goal of maintaining genetic diversity across biomes to preserve the evolutionary potential of the species.

Overall, the results reinforce the need to continue to build genomic resources for native South American species. Broader SNP discovery, genome-enabled analyses, and integrated molecular approaches will provide a stronger foundation for conservation planning and sustainable use, supporting the long-term preservation of ecologically and economically important native tree species.

## Figures and Tables

**Table 1:** Characteristics of ex situ conservation progeny tests installed at the Teaching, Research, and Extension Farm, Ilha Solteira Faculty of Engineering (FEPE/FEIS/UNESP), Selvíria, Mato Grosso do Sul, Brazil.

Population	Code	Biome	Installation
Petrolina—PE	PE	Caatinga dry forest	12/1992
Seridó—RN	SE	Caatinga dry forest	04/1997
Bauru—SP	BU	Brazilian Savanna	12/1987
Cuiabá—MT	CB	Brazilian Savanna	01/2010
Itarumã—GO	IT	Brazilian Savanna	06/2004
Selvíria—MS	SV	Brazilian Savanna-Atlantic Forest Ecotone (BAE)	12/1987
Paulo de Faria—SP	PF	Atlantic Forest	04/1997
Ribeirão Preto—SP	RP	Atlantic Forest	10/2006
Aquidauana—MS	AQ	Pantanal Wetland	12/2010
Corumbá—MS	CO	Pantanal Wetland	11/2023
Miranda—MS	MI	Pantanal Wetland	11/2023

**Table 2:** Environmental characteristics of the Atlantic Forest, Brazilian Savanna, Caatinga, Pantanal, and the Cerrado-Atlantic Forest ecotone (BAE) biomes, including average temperature, precipitation, elevation, soil characteristics, and other relevant characteristics.

Biome	Average Temp. (°C)	Precipitation (mm)	Elevation (m)	Soil	Key Characteristics	References
Atlantic Forest	20 to 26	1200 to 2200	0 to 2000	Moist, shallow, not naturally fertile, but rich in humus	High biodiversity; dense perennial vegetation with the presence of epiphytes; humid climate with well-distributed rainfall throughout the year.	[105]
Brazilian Savanna	22 to 33	1200 to 1800	300 to 1600	Acidic, nutrient-poor, with high water infiltration capacity	Seasonal tropical climate with a well-defined dry season; vegetation composed of grasses, shrubs and small trees with deep roots; natural occurrence of fires.	[106]
Caatinga	25 to 30	500 to 700	0 to 500	Shallow, stony, with little organic matter; relatively fertile	Semi-arid climate with long periods of drought; drought-adapted vegetation, including cacti and small shrubs; fauna adapted to arid conditions.	[107]
Ecotone (BAE)	20 to 24	1200 to 1800	300 to 800	Mixed, varying between soil characteristics of the Cerrado and the Atlantic Forest	Transition area with a mosaic of vegetation from the Cerrado and the Atlantic Forest; high biodiversity due to the overlap of species from both biomes; sensitive to environmental changes due to its ecological complexity.	[108]
Pantanal	22 to 32	800 to 1500	80 to 150	Alluvial soils, rich in nutrients, subject to periodic flooding	Largest floodplain in the world; alternates between periods of flood and drought; high biodiversity with the presence of aquatic and terrestrial species adapted to seasonal floods.	[109,110]

## Data Availability

All data supporting the results may be available on request and may be freely accessed for research and verification purposes.

## Supplementary Materials

The following supporting information can be downloaded at: https://www.mdpi.com/article/10.3390/plants15101505/s1, Supplementary Data S1: Estimation of private alleles in *M. urundeuva* from the biomes of the species occurrence in Brazil. Populations: Atlantic Forest: Paulo de Faria (PF), Ribeirão Preto (RP). Brazilian Savanna: Bauru (BU), Cuiabá (CB), Itarumã, (IT). Brazilian Savana-Atlantic Forest Transition (BAT): Active Germplasm Bank (BAG), Selvíria (SV). Caatinga dry forest: Petrolina (PE), Seridó (SE). Pantanal Wetlands: Aquidauana (AQ), Corumbá (CO), Miranda (MI). Supplementary Data S2: Estimation of allelic richness in *M. urundeuva* from the biomes of the species occurrence in Brazil. Populations: Atlantic Forest: Paulo de Faria (PF), Ribeirão Preto (RP). Brazilian Savanna: Bauru (BU), Cuiabá (CB), Itarumã, (IT). Brazilian Savana-Atlantic Forest Transition (BAT): Active Germplasm Bank (BAG), Selvíria (SV). Caatinga dry forest: Petrolina (PE), Seridó (SE). Pantanal Wetlands: Aquidauana (AQ), Corumbá (CO), Miranda (MI). Supplementary Data S3: Estimation of observed and expected heterozygosity in *M. urundeuva* from the biomes of the species occurrence in Brazil. Populations: Atlantic Forest: Paulo de Faria (PF), Ribeirão Preto (RP). Brazilian Savanna: Bauru (BU), Cuiabá (CB), Itarumã, (IT). Brazilian Savana-Atlantic Forest Transition (BAT): Active Germplasm Bank (BAG), Selvíria (SV). Caatinga dry forest: Petrolina (PE), Seridó (SE). Pantanal Wetlands: Aquidauana (AQ), Corumbá (CO), Miranda (MI). Supplementary Data S4: Correlation by principal component analysis (PCA) in *M. urundeuva* from the biomes of the species occurrence in Brazil. Populations: Atlantic Forest: Paulo de Faria (PF), Ribeirão Preto (RP). Brazilian Savanna: Bauru (BU), Cuiabá (CB), Itarumã, (IT). Brazilian Savana-Atlantic Forest Ecotone (BAE): Active Germplasm Bank (BAG), Selvíria (SV). Caatinga dry forest: Petrolina (PE), Seridó (SE). Pantanal Wetlands: Aquidauana (AQ), Corumbá (CO), Miranda (MI). Supplementary Data S5: Isolation analysis by distance by the Mantel test. Significant clustering between 0-1000km (comparison of genetic distances between central-western and southeastern populations of Brazil) and between above ~1250km-2500km distance (comparison between northeastern populations of Brazil). Supplementary Data S6: AMOVA (Analysis of Molecular Variance) for *M. urundeuva* populations from biomes of the species occurrence in Brazil. Supplementary Data S7: Climatic variables (PCoA) in *M. urundeuva* from the biomes of the species occurrence in Brazil. Populations: Atlantic Forest: Paulo de Faria (PF), Ribeirão Preto (RP). Brazilian Savanna: Bauru (BU), Cuiabá (CB), Itarumã, (IT). Brazilian Savana-Atlantic Forest Transition (BAT): Active Germplasm Bank (BAG), Selvíria (SV). Caatinga dry forest: Petrolina (PE), Seridó (SE). Pantanal Wetlands: Aquidauana (AQ), Corumbá (CO), Miranda (MI).

## Abbreviations

The following abbreviations are used in this manuscript:

AGB	Active Germplasm Bank
AFLP	Amplified Fragment Length Polymorphism
AMOVA	Analysis of Molecular Variance
BAG	Rosana
BU	Bauru
CB	Cuiabá
CO	Corumbá
CTAB	Cetyltrimethylammonium Bromide
DAPC	Discriminant Analysis of Principal Components
ddRADseq	Double Digest Restriction Associated DNA Sequencing
FEIS	Ilha Solteira School of Engineering
FEPE	Teaching, Research, and Extension Farm
FST	Wright’s Fixation Coefficient
He	Expected heterozygosity
Ho	Observed heterozygosity
IT	Itarumã
MI	Miranda
NGS	Next-Generation Sequencing
PCA	Principal Component Analysis
PCoA	Principal Coordinates Analysis
PE	Petrolina
PF	Paulo de Faria
RADseq	Restriction Digest Associated DNA Sequencing
RAPD	Random Amplified Polymorphic DNA
RDA	Redundancy Analysis
RP	Ribeirão Preto
SE	Seridó
SNPs	Single Nucleotide Polymorphisms
SV	Selvíria
TBE buffer	Tris/Borate/EDTA
UNESP	São Paulo State University
