# Supplementary material for: Assessing Genetic Divergence and Adaptive Potential of Aroeira (Myracrodruon urundeuva Allemão LC, Anacardiaceae) Across Brazilian Biomes
Source: Plants (Basel). 2026 May 15;15(10):1505. doi: 10.3390/plants15101505 (PMC13210503; doi:10.3390/plants15101505)
Supplement: Supplementary file 1 [file plants-15-01505-s001.zip › plants-4022301-supplementary.pdf]

Assessing Genetic Divergence and Adaptive Potential in Aroeira (*Myracrodruon urundeuva* Allemão LC, Anacardiaceae) across Brazilian Biomes

Marcelo Augusto Mendes Alcantara [1,2,†], Bruno Cesar Rossini [3,4,*,†], Marcela Aparecida de Moraes Silvestre [1], Romain Guyot [5], Andrea Garavito [6,7], Patricia Ferreira Alves [8], Diego Peres Alonso [4], Paulo Eduardo Martins Ribolla [1,4], Mario Luiz Teixeira de Moraes [8] and Celso Luis Marino [1,4]

[1] Department of Genetics, Microbiology and Immunology, Institute of Biosciences, São Paulo State University (Unesp), Botucatu 18618-689, São Paulo, Brazil
[2] Department of Forestry & Environmental Resources, College of Natural Resources, North Carolina State University, Raleigh 27607, North Carolina, United States
[3] Department of Bioprocesses and Biotechnology, School of Agricultural Sciences, São Paulo State University (Unesp), Botucatu 18610-034, São Paulo, Brazil
[4] Institute for Biotechnology, São Paulo State University (Unesp), Botucatu 18607-440, São Paulo, Brazil
[5] UMR DIADE , Research Institute for Development (IRD), University of Montpellier, CIRAD, 34394 Montpellier, France
[6] CIRAD, UMR AGAP Institut, F-34398 Montpellier, France
[7] UMR AGAP Institut, CIRAD, INRAE, Institut Agro, University of Montpellier, F-34398 Montpellier, France
[8] Department of Crop Sciences, Food Technology and Socioeconomics, School of Natural Sciences and Engineering, São Paulo State University (Unesp), Ilha Solteira 15385-007, São Paulo, Brazil
* Correspondence author: bruno.rossini@unesp.br
† These authors contributed equally to this work.

Supp. Data 1. Estimation of private alleles in *M. urundeuva* from the biomes of the species occurrence in Brazil. Populations: Atlantic Forest: Paulo de Faria (PF), Ribeirão Preto (RP). Brazilian Savanna: Bauru (BU), Cuiabá (CB), Itarumã, (IT). Brazilian Savana-Atlantic Forest Transition (BAT): Active Germplasm Bank (BAG), Selvíria (SV). Caatinga dry forest: Petrolina (PE), Seridó (SE). Pantanal Wetlands: Aquidauana (AQ), Corumbá (CO), Miranda (MI).

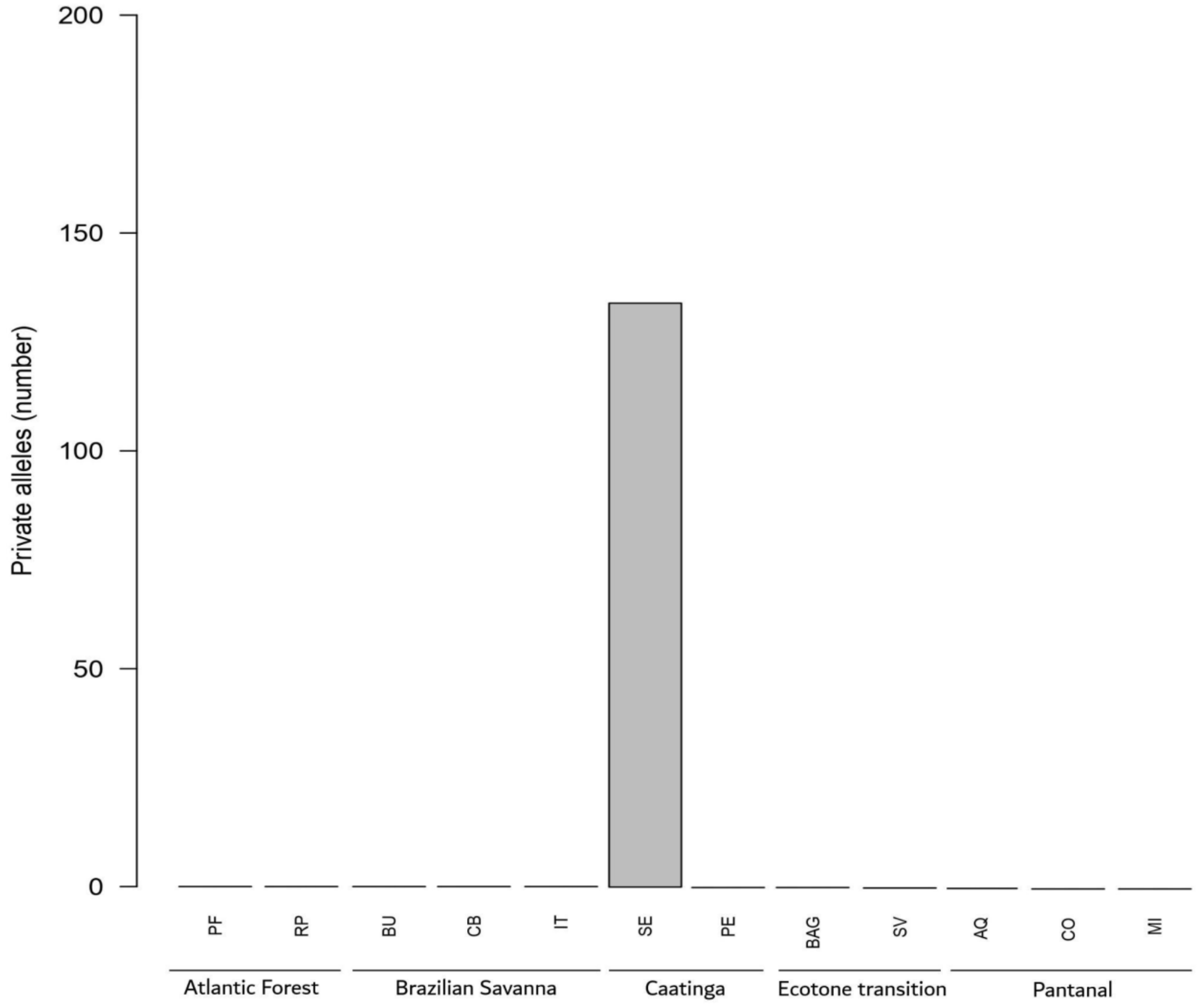

Supp. Data 2. Estimation of allelic richness in *M. urundeuva* from the biomes of the species occurrence in Brazil. Populations: Atlantic Forest: Paulo de Faria (PF), Ribeirão Preto (RP). Brazilian Savanna: Bauru (BU), Cuiabá (CB), Itarumã, (IT). Brazilian Savana-Atlantic Forest Transition (BAT): Active Germplasm Bank (BAG), Selvíria (SV). Caatinga dry forest: Petrolina (PE), Seridó (SE). Pantanal Wetlands: Aquidauana (AQ), Corumbá (CO), Miranda (MI).

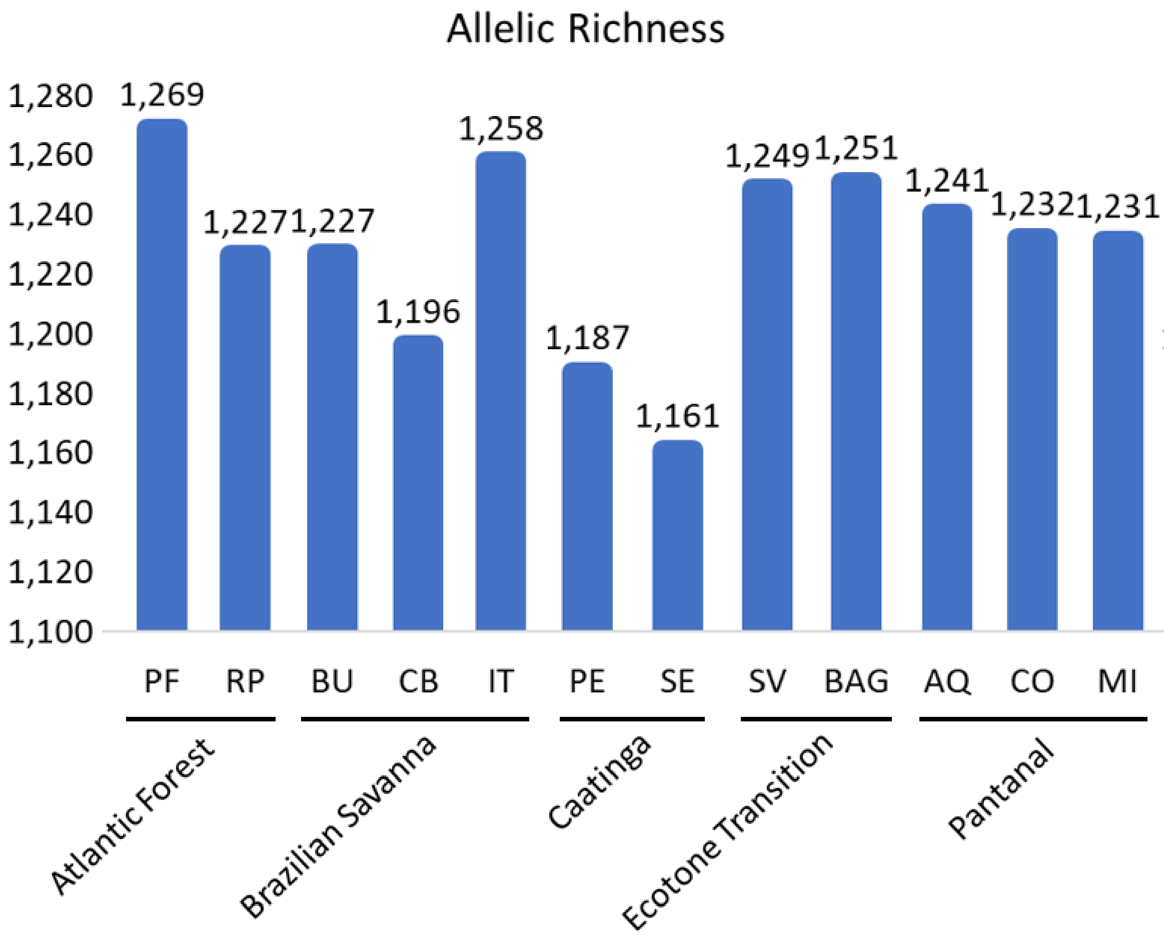

Supp. Data 3. Estimation of observed and expected heterozygosity in *M. urundeuva* from the biomes of the species occurrence in Brazil. Populations: Atlantic Forest: Paulo de Faria (PF), Ribeirão Preto (RP). Brazilian Savanna: Bauru (BU), Cuiabá (CB), Itarumã, (IT). Brazilian Savana-Atlantic Forest Transition (BAT): Active Germplasm Bank (BAG), Selvíria (SV). Caatinga dry forest: Petrolina (PE), Seridó (SE). Pantanal Wetlands: Aquidauana (AQ), Corumbá (CO), Miranda (MI).

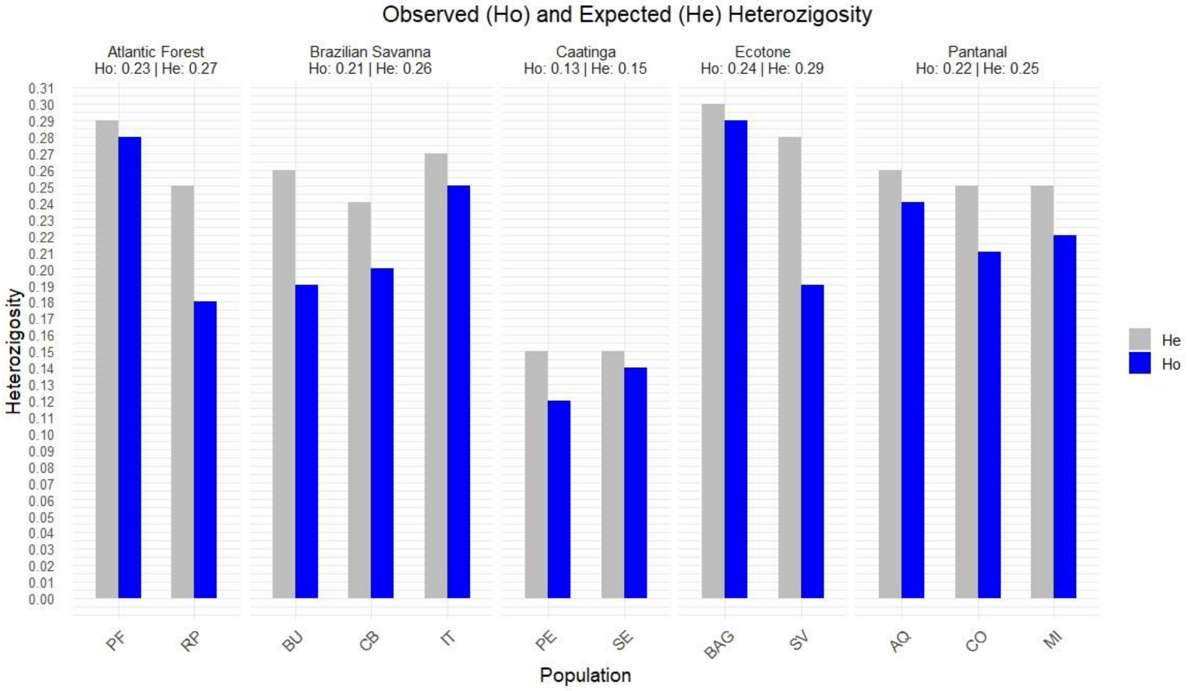

Supp. Data 4. Correlation by principal component analysis (PCA) in *M. urundeuva* from the biomes of the species occurrence in Brazil. Populations: Atlantic Forest: Paulo de Faria (PF), Ribeirão Preto (RP). Brazilian Savanna: Bauru (BU), Cuiabá (CB), Itarumã, (IT). Brazilian Savana-Atlantic Forest Ecotone (BAE): Active Germplasm Bank (BAG), Selvíria (SV). Caatinga dry forest: Petrolina (PE), Seridó (SE). Pantanal Wetlands: Aquidauana (AQ), Corumbá (CO), Miranda (MI).

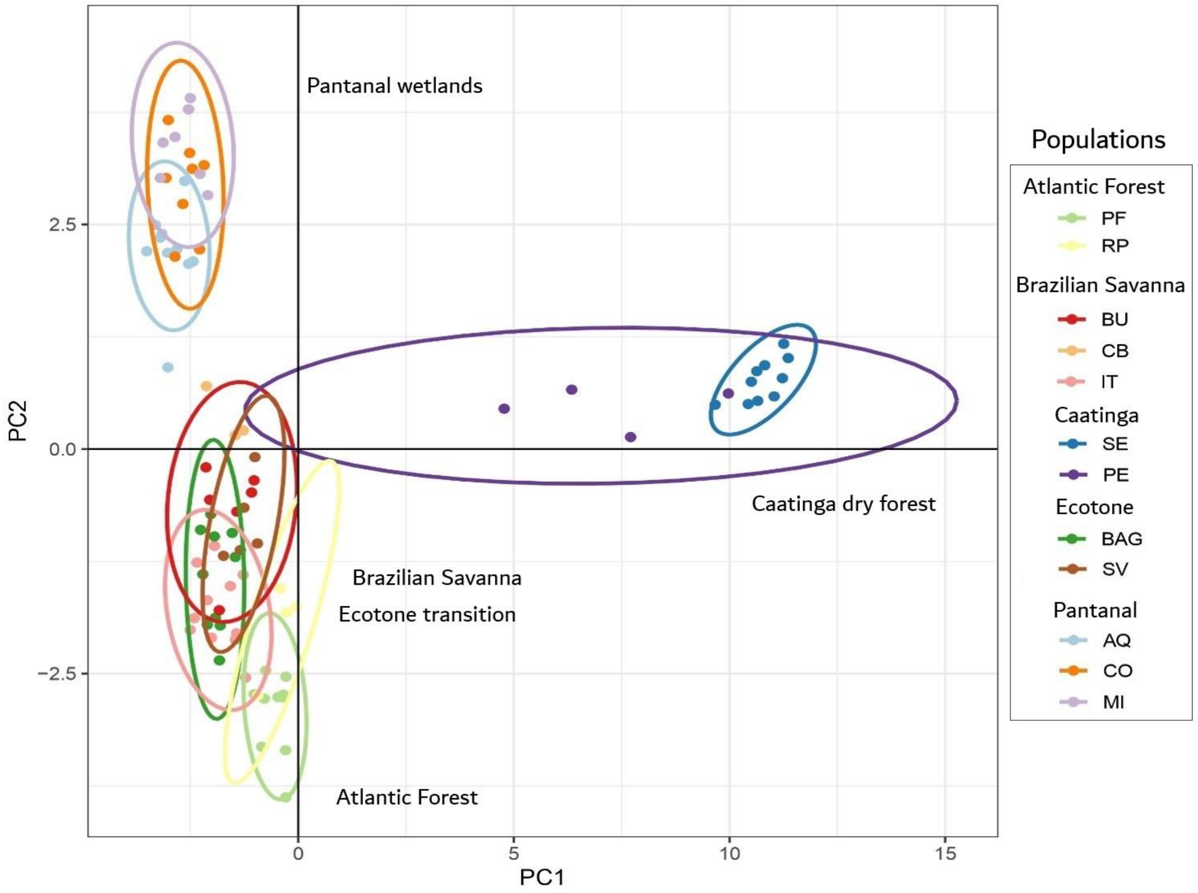

Supp. Data 5. Isolation analysis by distance by the Mantel test. Significant clustering between 0-1000km (comparison of genetic distances between central-western and southeastern populations of Brazil) and between above ~1250km-2500km distance (comparison between northeastern populations of Brazil).

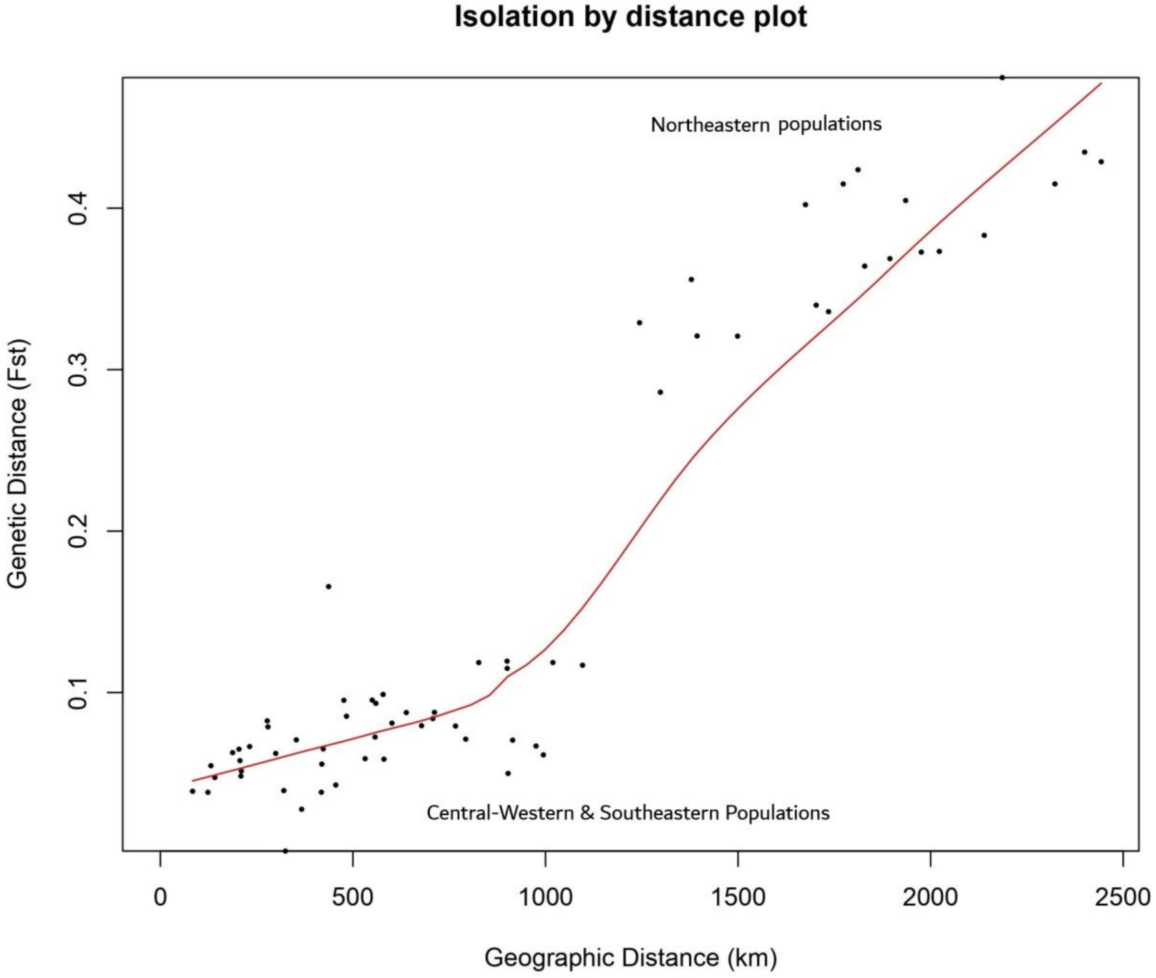

Supp. Data 6. AMOVA (Analysis of Molecular Variance) for *M. urundeuva* populations from biomes of the species occurrence in Brazil.

	Df	Sum Sq	Mean Sq
Between samples	11	7365.467	669.5879
Within samples	76	12956.283	170.4774
Total	87	20321.749	233.5833

	Sigma	%
Variations Between samples	68.94106	28.79521*
Variations Within samples	170.4774	71.20479
Total variations	239.4185	100
Based on 999 replicates		
* p-value:	0.001	

Supp. Data 7. Climatic variables (PCoA) in *M. urundeuva* from the biomes of the species occurrence in Brazil. Populations: Atlantic Forest: Paulo de Faria (PF), Ribeirão Preto (RP). Brazilian Savanna: Bauru (BU), Cuiabá (CB), Itarumã, (IT). Brazilian Savana-Atlantic Forest Transition (BAT): Active Germplasm Bank (BAG), Selvíria (SV). Caatinga dry forest: Petrolina (PE), Seridó (SE). Pantanal Wetlands: Aquidauana (AQ), Corumbá (CO), Miranda (MI).

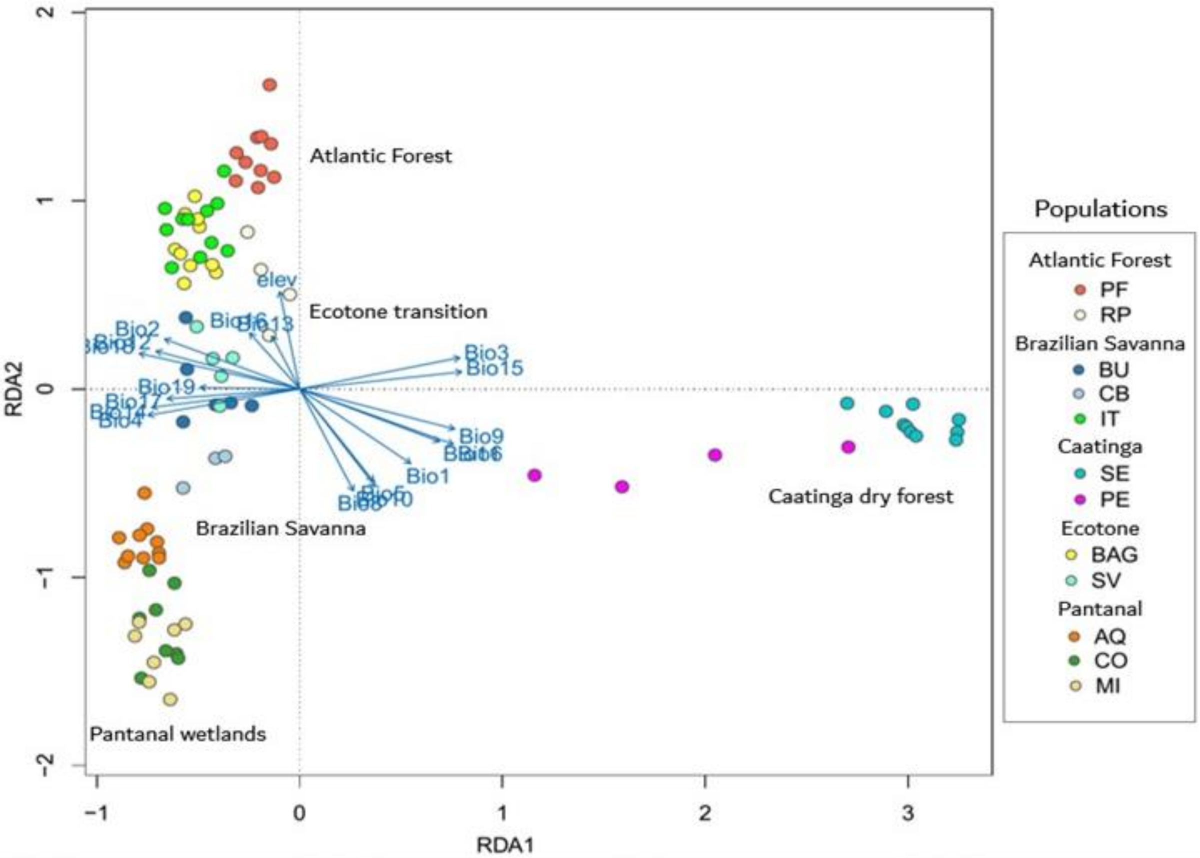

Code	Variable	Code	Variable
Bio1	Annual Mean Temperature	Bio11	Mean Temperature of Coldest Quarter
Bio2	Mean Diurnal Range (Mean of monthly) (max temperature - min temperature)	Bio12	Annual Precipitation
Bio3	Isothermality (BIO2 / BIO7 × 100)	Bio13	Wettest Month Precipitation
Bio4	Temperature Seasonality (Standard Deviation × 100)	Bio14	Driest Month Precipitation
Bio5	Max Temperature of Warmest Month	Bio15	Precipitation Seasonality (Coefficient of Variation)
Bio6	Min Temperature of Coldest Month	Bio16	Precipitation of Wettest Quarter
Bio7	Temperature Annual Range (BIO5 - BIO6)	Bio17	Precipitation of Driest Quarter
Bio8	Mean Temperature of Wettest Quarter	Bio18	Precipitation of Warmest Quarter
Bio9	Mean Temperature of Driest Quarter	Bio19	Precipitation of Coldest Quarter
Bio10	Mean Temperature of Warmest Quarter	elev	Relief Elevation
